# Food Fishes and Their Diseases

**Published:** 1880-10

**Authors:** Thomas F. DeVoe


### FOOD FISHES AND THEIR DISEASES.

By THOMAS F. DeVOE.

GOOD fish produces a wholesome, nutritious, and, in certain times of the year, a very cheap article of food. Some persons, however, have a prejudice against a fish diet, which they base on an assumption that it yields but little nutriment. The results of analysis of several kinds, however, prove that they contain nearly as much albuminous matter as the flesh of quadrupeds commonly used as food.

Fish are also said to be a flesh and muscle, not a fat producing aliment, as is obvious from the appearance of our seafaring population, who are usually spare, sinewy and strong, and free from those mountains of flesh and disease which characterize the prosperous beef-eaters or gormandizers.

In selecting fish which are fresh and fit to eat, they should appear firm and rigid, with bright red gills; but when stale, or having been caught several days, especially in warm weather, the flesh becomes soft and flabby; the gills have lost the red color; the fins are easily moved, and the eyes are usually dull and opaque; the fish is then in a state of decay and unfit for food.

We seldom find fresh fish of any kind which are unfit to be eaten, except in cases where they have been poisoned or infected with some sort of malaria, that taints the flesh, and gives it, even after it has been cooked, a very offensive odor, when no doubt it would cause sickness, and death, perhaps, if eaten.

Several instances of fish poisoning have occurred, both in the city of New York and other places within my recollection. A case in the family of Mr. Amos Jones, of No. 219 Sullivan street, New York, in 1854, who purchased shad at a fish-stand or wagon, and had them cooked; they were eaten by his family of nine persons, after which all were taken sick. The wife of Mr. Jones was taken with convulsions and died three days after; two others of the family remained very sick several days, but eventually all the others got well. A post-mortem examination was held on the body by Drs. Uhl, Williams, and Guernelly, who found the stomach very much inflamed.

In the same year, near Baltimore, in the month of September, large numbers of dead fish of all kinds floated on shore near Federal Hill, and were so numerous that steam tugs and scows were put in requisition to carry them away, and preserve the health of the neighborhood. This mortality among the fish was supposed to be the gaseous matter exuding from the Chemical Works, that occasioned this destruction; or it may have been some maratime epidemic. But the fact was remarkable, and the only wonder was, that some of those first caught, and sent to market, did not infect those who eat them.

There was also a great mortality among the fish in the Passaic River, in the month of June, 1877, where for miles above Patterson, their putrefying carcases lined the banks on both sides of the river, in countless numbers. When first attacked, the sides and back of the fish were covered with bright red spots, about the size of a three-cent piece. There was also a thin blue, or greyish film that covered the head. The fish would rush rapidly through the water, as if undergoing terrible pain. In a short time their struggles grew feebler, and death soon ended their torture.

An oyster epidemic also took place in New York, in the month of October, 1854, when several prominent citizens died, and others were very sick, in consequence of eating oysters.

Among the deaths were Edwin Williams, ex-Alderman Smith, James Foster, Morris M. Davidson, John H. Cornell, and John Monell. At the same time there was much sickness and disease in Baltimore and other places. It appears evident that oysters at that period were exceedingly unhealthy, and various causes were assigned for their poisonous quality; some attributed it to drought; others thought the oysters were taken up during their spawning-time; and an old fisherman said he thought both oysters and crabs were unsafe to be eaten, being, possessed of some impure or poisoning substance; another wrote that it was a singular fact that the oysters taken from the Potomac River, near Baltimore, proved to be deleterious to health. Many persons, after having partaken of them in a raw state, were violently attacked with cramps, colic, cholera-morbus etc.

